# When people live with multiple chronic diseases: a collaborative approach to an emerging global challenge

**Published:** 2011-09-20

**Authors:** Nuria Toro

**Affiliations:** O+berri (Basque Institute for Healthcare Innovation), Spain, E-mail: toro@bioef.org

Polypathology is one of the most pressing challenges in the present and future management of chronic disease. This book is an edited collection of essays that cover both conceptual and research dimensions of polypathology. It is a living document that is continuously evolving as readers are invited to make their own contributions to the discussion on the web (www.opimec.org). Although the book is not specifically devoted to integrated care, this theme is at the core of several chapters.

There are 10 co-authored chapters and an epilogue on a range of aspects related to multiple chronic diseases. Even though the content and length of each chapter vary, all follow the same structure and contain the same sections, making it very easy to read. The book is available both in English and Spanish.

Now let us consider each chapter in some detail.

Chapter 1 starts by recognising the emergence of polypathology, a new phenomenon that must be carefully analysed and approached due to its huge impact in health, economic and social spheres. The book, co-created by more than 500 contributors, intends to shed light on poorly-understood areas related to polypathology and so this sets the context for the following chapters.

Chapter 2 is a very stimulating introductory chapter that highlights the lack of an accepted terminology to identify, characterise and code what happens to people who live with multiple chronic diseases. It analyses in great depth the plethora of terms that are used across the world and their implications for management and with respect to integration. This review may be very useful for readers.

Chapter 3 provides a very comprehensive analysis of chronic disease prevention and health promotion, without considering the perspective of integration. After recognising that there is little evidence to indicate the best approaches for the prevention of polypathology, the authors go on to discuss in detail the prevention of individual chronic conditions. Although it addresses highly topical issues such as the polypill, it deviates in general terms from the core subject, that is, polypathology, offering a rather broad discourse on prevention that can also be found in the mainstream chronic disease literature. Finally, the question on how to tackle prevention of multiple chronic diseases remains unanswered.

Chapter 4 is one of the cornerstones of the book. It outlines the most relevant international models to improve the health of those living with two or more chronic conditions. Starting from the most generic chronic disease management models, such as the chronic care model, authors go on to describe the models, tools and programmes that can be applied to complex patients based on integration. What makes this chapter so appealing is the well-judged balance between theory and evidence that can provide the reader with an understanding of some key elements for the successful management of multiple chronic diseases. At the end of the chapter, authors call for new paradigms to implement the needed change to transform health systems, an interesting reflection that is important for decision makers and managers alike.

Chapter 5 examines various strategies and types of programme for patient education and self-management support. The rationale behind the chapter is clear: self-management in chronic patients is expected to improve outcomes and reduce healthcare costs. It provides an exhaustive conceptual framework for different approaches to self-management. However, little is said concerning the effectiveness of these programmes and what effect they may have on quality of care and costs. Moreover, as occurred in chapter 3, the authors do not focus on the use of these tools within the context of multiple chronic diseases, but rather offer a general discourse on the broader theme of chronic disease. Finally, due to the nature of the topic that is being dealt with, the chapter has no real relevance to integrated care.

Chapter 6 should be another core chapter of the book as it addresses the need for integrated and comprehensive models of care that meet the challenge of managing complex diseases. Authors start by calling for integrated approaches between health and social care but the international experience with these models is not discussed. Then, the chapter provides an interesting perspective on primary care as the key player of the health system for dealing with complex patients and wisely warns against transplanting international models to local settings without adaptation. In this context, the concept of process reengineering is dealt with in a rather superficial way; more detail would be required to understand how it could be applied in practice in health systems. Overall, in the reviewer's opinion, this chapter is somewhat over optimistic in its approach, since key problems, such as the prevailing hospital-centric culture and barriers to integration, are overlooked and overly simple solutions, such as process reengineering, are provided to an issue as complex as integration in which culture and power has so much influence.

Chapter 7 is quite relevant to integration. It is argued that palliative and supportive care must be carried out through an integrated approach that focuses both on psychological and spiritual aspects of the patient’s condition and on the integration and coordination of different roles through multidisciplinary teams. It is a comprehensive discussion that considers present and future possibilities in the development of comprehensive palliative care for patients with multiple complex diseases.

Chapter 8 provides an analysis of alternative medical philosophies and practices. In the reviewer's opinion, it deviates from the scientific approach followed by the rest of the book; as it is recognised, most treatments are recommended on the basis of opinion rather than research and, in many cases, efficacy has not been proven. Accordingly, the topic of alternative medicine needs to be considered. Nonetheless, references in the chapter to a need for debate on demedicalisation should not be overlooked. Finally, the reader should take into account that this chapter has little relevance to integration other than the fact that it advocates integrating conventional and traditional practices as part of the solution to multiple complex diseases.

Chapter 9 focuses on the socioeconomic implications of multiple chronic diseases. It is a well-documented chapter, full of figures and statistical data, and stresses the need for health promotion and prevention and the implementation of innovative models for complex disease management. Though not specifically addressed, this chapter contains some strong rationale for integration.

Chapter 10 examines the role of genomics, robotics, IT and nanotechnology in an attempt to illustrate their potential contribution to dealing with multiple complex diseases. The reader should not expect even a mention of integrated care. On the other hand, it may be appealing for enthusiasts of “sci-fi speculation” who would like to know more about the ways in which technology may have an impact on our lives in the future.

In conclusion, this book is not a book on integration but the features of multiple chronic diseases mean that integrated care is identified as a suitable approach for such patients. Since polypathology is a multidimensional concept, certain chapters address integrated care explicitly, while others do not.

It is a book mostly aimed at raising awareness on polypathology. It looks for a general understanding on multiple chronic diseases rather than for specific answers for the questions it poses. In fact, many questions may remain unanswered and an explicit call for further research and evidence is repeatedly done.

It is particularly relevant for health policy makers, managers and health professionals looking for a global approach on multiple chronic diseases and its implications for the socioeconomic aspects of healthcare and health management.

